# Author Correction: Metagenome-assembled genomes provide new insight into the microbial diversity of two thermal pools in Kamchatka, Russia

**DOI:** 10.1038/s41598-020-60390-y

**Published:** 2020-02-21

**Authors:** Laetitia G. E. Wilkins, Cassandra L. Ettinger, Guillaume Jospin, Jonathan A. Eisen

**Affiliations:** 1 Department of Environmental Sciences, Policy & Management, University of California, Berkeley, CA 94720 USA; 2 Genome Center, University of California, Davis, CA 95616 USA; 3 Department of Evolution and Ecology, University of California, Davis, CA 95616 USA; 4 Department of Medical Microbiology and Immunology, University of California, Davis, CA 95616 USA

Correction to: *Scientific Reports* 10.1038/s41598-019-39576-6, published online 28 February 2019

A supplementary file containing Tables S1-16 was omitted from the original version of this Article. This has been corrected in the HTML version of the Article; the PDF version was correct at time of publication.

