# Supplementary material for: Metagenome-assembled genomes provide new insight into the microbial diversity of two thermal pools in Kamchatka, Russia
Source: Sci Rep. 2019 Feb 28;9:3059. doi: 10.1038/s41598-019-39576-6 (PMC6395817; doi:10.1038/s41598-019-39576-6)
Supplement: Supplementary file 1 — Supplementary Materials after revision [file 41598_2019_39576_MOESM1_ESM.pdf]

Supplementary Materials:

Metagenome-assembled genomes provide new insight into the microbial diversity of two thermal pools in Kamchatka, Russia

Laetitia G. E. Wilkins[#*], Cassandra L. Ettinger[#], Guillaume Jospin & Jonathan A. Eisen

# These authors contributed equally

*Corresponding author: Laetitia G. E. Wilkins, lgwilkins@ucdavis.edu
Genome Center, 451 Health Science Drive, University of California, Davis
Davis, CA 95616, USA
+1 530-752-2237

Supplementary Methods:

Clone library creation and Sanger sequencing

Clone libraries were created from the DNA at The Institute for Genomic Research (TIGR) in 2005 by Lakshmi Viswanathan using what were then standard library construction protocols [1]. In summary, genomic DNA was sheared using nebulization, and then after agarose gel electrophoresis, DNA of a specific targeted size was excised, purified, and cloned into the pHOS2 plasmid vector. For the KAM sample the library insert was ~ 7 kb. For the ZAV sample the library insert was ~ 9 kb in size. Then, sequencing reads were generated from the libraries by first transforming *Escherichia coli*, picking colonies, purifying plasmids, and then doing paired-end sequencing using the plasmid primers on both sides of the insert to prime the reactions. Sequencing was carried out in 2006 at the Joint Technology Center (https://www.jcvi.org/tigr-ibea-and-tcag-create-new-high-throughput-genomic-sequencing-facility) on ABI 3730 sequencers.

Solexa sequencing

Sequencing library preparations for Solexa3 84 bp paired-end sequencing were performed by the UC Davis Genome Center DNA Technologies Core Facility (http://dnatech.genomecenter.ucdavis.edu/), where the samples were sequenced on two lanes.

Construction of the microbial phylogenetic tree

The genomes used by Hug *et al*. were downloaded using the script 'ncbi_download.py' from Christopher T. Brown's GitHub page (https://github.com/christophertbrown/bioscripts/tree/master/ctbBio) using Table S1 from Hug *et al*. as queries. The automated download of genomes from NCBI yielded 2,991 genomes. Ninety genomes were downloaded manually based on identifiers in Hug *et al*.'s supplementary Table S1 [2].

PhyloSift builds an alignment of the concatenated sequences for a set of core markers for each taxon. For more information about the available core marker gene sets see Darling *et al*. 2014 (updated last on 2018-02-12). We used 37 of these single-copy marker genes. The amino acid alignment of these 37 concatenated genes was trimmed using trimAl v.1.2 [3]. Columns with gaps in more than 5% of the sequences were removed, as well as taxa with with less than 75% of the concatenated sequences. MAGs from ARK and ZAV that did not meet this threshold were manually kept in the alignment. The final alignment [4] comprised 3,240 taxa (Supplementary Table S3) and 5,459 amino acid positions. This alignment was then used to build a new phylogenetic tree in RAxML v. 8.2.10 on the CIPRES Science Gateway web server [5]. First, we searched for the best protein substitution model of the alignment with its empirical base frequencies using the Bayesian Information Criterion (BIC) within RAxML. Then, this substitution model; *i.e*, LG plus CAT (after Le and Gascuel) [6], was used to infer a phylogenetic tree. We chose the rapid bootstrapping algorithm (flag -f a) to find the best scoring maximum likelihood tree with 10 starting trees in one run with the number of

bootstraps automatically determined (MRE-based bootstopping criterion). One hundred fifty bootstrap replicates were conducted. The full tree inference required 2,236 computational hours on the CIPRES supercomputer (raw tree file in newick format available on figshare [7]). The Interactive Tree Of Life website iTOL was used to finalize and polish the tree for publication [8]. Taxa from the genera *Buchnera* (Enterobacteriaceae, endosymbiont of aphids) and *Mycoplasma* were removed manually from the tree during visual inspection due to them being at the end of extraordinarily long evolutionary branches.

PhyloSift Markers used in this study

Ribosomal protein S2 rpsB, S10 rpsJ, L1 rplA, L22, L4/L1e rplD, L2 rplB, S9 rpsI, L3 rplC, L14b/L23e rplN, S5, S19 rpsS, S7, L16/L10E rplP, S13 rpsM, L15, L25/L23, L6 rplF, L11 rplK, L5 rplE, S12/S23, L29, S3 rpsC, S11 rpsK, L10, S8, L18P/L5E, S15P/S13e, S17, S13 rplM, L24; and translation initiation factor IF-2, metalloendopeptidase, phenylalanyl-tRNA synthetase beta subunit, phenylalanyl-tRNA synthetase alpha subunit, tRNA pseudouridine synthase B, Porphobilinogen deaminase, and ribonuclease HII; i.e., PhyloSift markers DNGNGWU00001 - DNGNGWU00040 without DNGNGWU00004, DNGNGWU00008 and DNGNGWU00038.

For a detailed walk-through of the analyses, please refer to the associated Jupyter notebooks for ZAV[9] and for ARK[10].

NCBI's Foreign Contamination Screens

NCBI's Foreign Contamination Screens is not a publicly available service yet. It is an integrated part of the submission.

The purpose of the foreign contamination screens is to identify contaminating sequences that may be present for artificial reasons or for biological reasons. Artificial reasons include cloning artifacts (vector, linker/adaptor/primer, E. coli host DNA), contamination in the lab with human sequence, mixing of samples or sequencing runs with other organisms, and bacterial insertion sequences that have integrated into sequenced clones. Biological reasons include the presence of endosymbionts, infectious agents, or microbes residing on the surface of the organism or in the gut when the DNA prep was made.

The suite of foreign contamination screens uses BLAST to screen the submitted sequences against:

1. a common contaminants database that contains vector sequences, bacterial insertion sequences, E. coli and phage genomes

2. a database of adaptors linkers and primers

3. a database of mitochondrial genomes

4. the chromosomes of unrelated organisms

5. a database of ribosomal RNA genes

Suspect spans are re-BLASTed against:

1. the chromosomes of unrelated organisms

2. the chromosomes of related organisms

3. the NCBI nt BLAST database of nucleotide sequence from all traditional divisions of GenBank, EMBL, and DDBJ

4. the NCBI htgs BLAST database of sequences from the HTG division of GenBank, EMBL, and DDBJ

Results similar to those obtained by NCBI could be generated by running the screens as described below.

1. Common contaminant screen
===========================

Databases
----
a) File to screen for the common contaminants in eukaryotic sequences:

ftp://ftp.ncbi.nlm.nih.gov/pub/kitts/contam_in_euks.fa.gz

Contains the cloning artifacts that are likely to show up as contaminants across all eukaryotic species: vector sequences, E.coli genome, phage genomes, bacterial Insertion Sequences and transposons.

b) File to screen for the common contaminants in prokaryotic sequences:
ftp://ftp.ncbi.nlm.nih.gov/pub/kitts/contam_in_prok.fa

Contains phiX174.

These files need to be unzipped and the resulting FASTA sequence files formatted as BLAST databases using the makeblastdb program.

Programs

----

blastn and makeblastdb are contained in the blast+ package which can be installed following the instruction in the BLAST help documents.

"BLAST Command Line Applications User Manual": https://www.ncbi.nlm.nih.gov/books/NBK279671/

"Standalone BLAST Setup for Windows PC": https://www.ncbi.nlm.nih.gov/books/NBK52637/

"Standalone BLAST Setup for Unix": https://www.ncbi.nlm.nih.gov/books/NBK52640/

Execution

A BLAST search is run against either the contam_in_euks or contam_in_prok database, depending on the origin of the input sequences. The common contaminant BLAST results are filtered for hits over various length and percent identity cut-offs.

Command line:

a) for screening eukaryotic sequences:

```
blastn -query _input_fasta_sequences_ -db contam_in_euks -task megablast -word_size 28 -best_hit_overhang 0.1 -best_hit_score_edge 0.1 -dust yes -evalue 0.0001 -perc_identity 90.0 -outfmt "7 qseqid sseqid pident length mismatch gapopen qstart qend sstart send evalue bitscore" | awk '($3>=98.0 && $4>=50)||($3>=94.0 && $4>=100)||($3>=90.0 && $4>=200)'
```

OR with an intermediate file, these 2 commands:

```
blastn -query _input_fasta_sequences_ -db contam_in_euks -task megablast -word_size 28 -best_hit_overhang 0.1 -best_hit_score_edge 0.1 -dust yes -evalue 0.0001 -perc_identity 90.0 -outfmt "7 qseqid sseqid pident length mismatch gapopen qstart qend sstart send evalue bitscore" -out _out_file_
```

```
awk '($3>=98.0 && $4>=50)||($3>=94.0 && $4>=100)||($3>=90.0 && $4>=200)' _out_file_
```

b) for screening prokaryotic sequences:

blastn -query _input_fasta_sequences_ -db contam_in_prok -task megablast -word_size 28 -best_hit_overhang 0.1 -best_hit_score_edge 0.1 -dust yes -evalue 0.0001 -perc_identity 90.0 -outfmt "7 qseqid sseqid pident length mismatch gapopen qstart qend sstart send evalue bitscore" | awk '($3>=98.0 && $4>=50)||($3>=94.0 && $4>=100)||($3>=90.0 && $4>=200)'

OR with an intermediate file, these 2 commands:

blastn -query _input_fasta_sequences_ -db contam_in_prok -task megablast -word_size 28 -best_hit_overhang 0.1 -best_hit_score_edge 0.1 -dust yes -evalue 0.0001 -perc_identity 90.0 -outfmt "7 qseqid sseqid pident length mismatch gapopen qstart qend sstart send evalue bitscore" -out _out_file_

awk '($3>=98.0 && $4>=50)||($3>=94.0 && $4>=100)||($3>=90.0 && $4>=200)' _out_file_

2. Adaptor screen
=================
VecScreen (https://www.ncbi.nlm.nih.gov/tools/vecscreen/) is run against either the adaptors_for_screening_euks.fa database or adaptors_for_screening_proks.fa database, depending on the origin of the input sequences. Hits are filtered to retain only those matches that VecScreen classifies as "Strong" or "Moderate" (see: https://www.ncbi.nlm.nih.gov/tools/vecscreen/about/#Categories).

Databases

The adaptors_for_screening databases are available here:

ftp://ftp.ncbi.nlm.nih.gov/pub/kitts/adaptors_for_screening_euks.fa

ftp://ftp.ncbi.nlm.nih.gov/pub/kitts/adaptors_for_screening_proks.fa

These FASTA sequence files need to be formatted as BLAST databases using the makeblastdb program.

Programs

The VecScreen standalone program is available here:

ftp://ftp.ncbi.nlm.nih.gov/blast/demo/vecscreen

The script to filter the VecScreen results is here:
ftp://ftp.ncbi.nlm.nih.gov/pub/kitts/VSlistTo1HitPerLine.awk

Execution

Command line:

a) for screening eukaryotic sequences:

vecscreen -d adaptors_for_screening_euks.fa -f3 -i _input_fasta_sequences_ -o _vs_output_file_

b) for screening prokaryotic sequences:

vecscreen -d adaptors_for_screening_proks.fa -f3 -i _input_fasta_sequences_ -o _vs_output_file_

Filter out the "Weak" and "Suspect Origin" hits:
VSlistTo1HitPerLine.awk suspect=0 weak=0 _vs_output_file_ > _filtered_vs_output_file_

3. Mitochondrial genome screen
==============================

BLAST is used to screen the input sequences against a database of the mitochondrial genome sequences in the NCBI Reference Sequences (RefSeq) collection.

Database
----
ftp://ftp.ncbi.nlm.nih.gov/blast/db/FASTA/mito.nt.gz

This file needs to be unzipped and the resulting FASTA sequence file formatted as a BLAST database using the makeblastdb program.

Programs
----

blastn and makeblastdb are contained in the blast+ package (see above).

Execution

The BLAST hits to mitochondrial genomes are filtered for hits over 98.6% identity and at least 120 bases long.

```
blastn -query _input_fasta_sequences -db mito.nt -out % -task megablast -word_size 28 -best_hit_overhang 0.1 -best_hit_score_edge 0.1 -dust yes -evalue 0.0001 -perc_identity 98.6 -soft_masking true -outfmt 7 | awk '$4>=120' > _filtered_mito_output_file_
```

4. Ribosomal RNA screen
======================
Ribosomal RNA genes are the cause of many false positives because the include some segments that align to distantly related organisms. Segments that match rRNA genes are identified so that such segments are not reported as being foreign.

BLAST is used to screen the input sequences against a database of the rRNA gene sequences .

Database
----
ftp://ftp.ncbi.nlm.nih.gov/pub/kitts/rrna.gz

This file needs to be unzipped and the resulting FASTA sequence file formatted as a BLAST database using the makeblastdb program.

Programs
----
blastn and makeblastdb are contained in the blast+ package (see above).

Execution

The BLAST hits to rRNA genes are filtered for hits over 95% identity and at least 100 bases long.

```
blastn -query _input_fasta_sequences_ -db rrna -task megablast -template_length 18 -template_type coding -window_size 120 -word_size 12 -xdrop_gap 20 -no_greedy -best_hit_overhang 0.1 -
```

best_hit_score_edge 0.1 -dust yes -evalue 1E-9 -gapextend 2 -gapopen 4 -penalty -4 -perc_identity 95 -reward 3 -soft_masking true -outfmt 7 | awk '$4>=100' > _filtered_mito_output_file_

5. Foreign chromosome screen
============================

Screens for matches to chromosome sequences from foreign organisms. Foreign organisms are those that belong to a different taxonomic group compared to the organism whose sequences are being screened. The taxonomic groups are:

  arthropoda, chordata, other_metazoa,

  viridiplantae, fungi, other_eukaryota,

  bacteria, archaea, viruses_and_viroids

Databases

Our databases to detect cross-contamination detection are limited to assemblies that have been publicly released in GenBank/ENA/DDBJ and subsequently picked up by RefSeq. Genome centers can do better by augmenting these databases with additional genomes that they have sequenced but which are not yet represented in the RefSeq collection.

a) archaea

Query in Nucleotide :

archaea[porgn] AND srcdb_refseq[prop] AND biomol_genomic[prop] AND complete[prop]

b) bacteria

Query in Nucleotide :

bacteria[porgn] AND srcdb_refseq[prop] AND biomol_genomic[prop] AND complete[prop]

c) fungi

Query in Nucleotide :

fungi[porgn] AND srcdb_refseq[prop] AND biomol_genomic[prop] AND (NC_000000:NC_999999[pacc] OR AC_000000:AC_999999[pacc] OR (NT_000001:NT_999999999[pacc] AND ("chromosome 2L" OR "chromosome 2R" OR "chromosome 3L" OR "chromosome 3R")))

d) arthropoda

Query in Nucleotide :

arthropoda[porgn] AND srcdb_refseq[prop] AND biomol_genomic[prop] AND (NC_000000:NC_999999[pacc] OR AC_000000:AC_999999[pacc] OR (NT_000001:NT_999999999[pacc] AND ("chromosome 2L" OR "chromosome 2R" OR "chromosome 3L" OR "chromosome 3R")))

e) chordata

Query in Nucleotide :

chordata[porgn] AND srcdb_refseq[prop] AND biomol_genomic[prop] AND (NC_000000:NC_999999[pacc] OR AC_000000:AC_999999[pacc] OR (NT_000001:NT_999999999[pacc] AND ("chromosome 2L" OR "chromosome 2R" OR "chromosome 3L" OR "chromosome 3R")))

f) other_metazoa

Query in Nucleotide :

metazoa[porgn] NOT (arthropoda[porgn] OR chordata[porgn]) AND srcdb_refseq[prop] AND biomol_genomic[prop] AND (NC_000000:NC_999999[pacc] OR AC_000000:AC_999999[pacc] OR (NT_000001:NT_999999999[pacc] AND ("chromosome 2L" OR "chromosome 2R" OR "chromosome 3L" OR "chromosome 3R")))

g) viridiplantae

Query in Nucleotide :

viridiplantae[porgn] AND srcdb_refseq[prop] AND biomol_genomic[prop] AND (NC_000000:NC_999999[pacc] OR AC_000000:AC_999999[pacc] OR (NT_000001:NT_999999999[pacc] AND ("chromosome 2L" OR "chromosome 2R" OR "chromosome 3L" OR "chromosome 3R")))

h) other_eukaryota

Query in Nucleotide :

eukaryota[porgn] NOT (metazoa[porgn] OR fungi[porgn] OR viridiplantae[porgn]) AND srcdb_refseq[prop] AND biomol_genomic[prop] AND (NC_000000:NC_999999[pacc] OR AC_000000:AC_999999[pacc] OR (NT_000001:NT_999999999[pacc] AND ("chromosome 2L" OR "chromosome 2R" OR "chromosome 3L" OR "chromosome 3R")))

i) viruses_and_viroids

Query in Nucleotide :

(viruses[porgn] OR viroids[porgn]) AND srcdb_refseq[prop] AND biomol_genomic[prop] AND (NC_000000:NC_999999[pacc] OR AC_000000:AC_999999[pacc] OR (NT_000001:NT_999999999[pacc] AND ("chromosome 2L" OR "chromosome 2R" OR "chromosome 3L" OR "chromosome 3R")))

The FASTA sequence files resulting from these queries are formatted as nine BLAST databases using the makeblastdb program.

Execution

Repeats in the input FASTA sequences are soft-masked to lowercase using WindowMasker. Then BLAST hits over 98% identity are generated to the databases for the 8 taxonomic groups to which the organism being screened does not belong.

blastn -query _input_fasta_sequences_ -db _distant_organism_dbs_ -task megablast -word_size 28 -best_hit_overhang 0.1 -best_hit_score_edge 0.1 -dust yes -evalue 0.0001 -min_raw_gapped_score 100 -penalty -5 -perc_identity 98.0 -soft_masking true

6. First pass calls
===================
The following heuristic rules help to get rid of most false matches.
Process contaminant matches from 1

Contaminant matches from (1) are merged if they are from the same class of sequence (VECTOR, E.coli, IS, PHG) and they overlap or are separated by 50 bases or less.

If the total coverage of contaminant matches from (1) is >75% of the sequence length then flag the sequence as a contaminant to be excluded.

If the contaminant is classed as VECTOR, E.coli, IS:.*, PERM:.* or PHG:* and the contaminant location is within 100 bases of the the start or end of the sequence (or gap is the sequence is not contiguous), or within 100 bases of another contaminant match that is at an end, flag the contaminant span for trimming.

If the contaminant is one of the above, and the match is longer than 700 bases flag the contaminant span for trimming.

Other matches may be false alarms. Treat them as suspect spans and reBLAST the hit span plus 10 Kbp of flanking sequence on each side against nr, HTGS, related and unrelated chromosomes (as described below).

Process contaminant matches from 2

Flag all adaptor spans for trimming.

Process mitochondrion matches from 3

If the total coverage of mitochondrial matches from (3) is >75% of the sequence length then flag the sequence as being mitochindrial sequence to be excluded.

Process unrelated chromosome matches from 5

Ignore any matches to chromosomes from unrelated organisms that lie with a region identified as being rRNA genes from (4) (the spans matched in 4 plus 100 bases on both sides). These are likely to be false matches.

Treat other matched spans as suspect and reBLAST the hit span plus 10 Kbp of flanking sequence on each side against nr, HTGS, related and unrelated chromosomes

ReBLAST against nr, HTGS, related and unrelated chromosomes

Spans identified a contamination suspects in the first pass, plus 10 Kbp of flanking sequence on each side (up to the end of the contig), are BLASTed against nr, HTGS, related and unrelated chromosomes to generate additional data for calling contaminants to be excluded or trimmed.

Databases
----

chromosome databases (a) to (i) from (5) above.

ftp://ftp.ncbi.nlm.nih.gov/blast/db/nt.*.tar.gz
ftp://ftp.ncbi.nlm.nih.gov/blast/db/htgs.*.tar.gz

Execution

The suspect spans are BLASTed against each of the 10 databases.

blastn -query _suspect_spans_plus_flanks_ -db _reblast_db_ -task megablast -dust yes -evalue 1E-9 -searchsp 1000000000 -perc_identity 98.0 -soft_masking true

Processing the reBLAST matches

Automatically exclude sequence contigs that meet all the following criteria:

60% of length covered with foreign hits, or less than 200 bp that are NOT covered

Each contributing hits must be 100 bp or longer with identity >= 98%

The best match to chromosomes from unrelated organisms is longer than the best match to chromosomes from the related organism group

Legends for the supplementary Excel tables:

Table S1: Summary statistics of quality filtering and metagenomic assembly.
Arkashin Schurf (ARK) and Zavarzin Spring (ZAV) were assembled separately resulting in two metagenomes. Bp = basepairs, nt = nucleotides.

Table S2: Summary of individual MAG assembly statistics, quality measurements and taxonomic inferences.
Here for each metagenome-assembled genome (MAG), we report basic assembly statistics, including total assembly size, contig N50 and GC content, quality measurements, including completion, contamination and strain heterogeneity, and taxonomic inference.

Table S3: Genomes used in tree, their names, and NCBI accession numbers.
Table with all the taxa that were used to construct the tree in Fig. 2 (genomes of archaea and bacteria used to infer a tree in Hug *et al.* 2016); MAGs isolated from two hot springs in the Uzon Caldera, Kamchatka, Russia; and genomes of taxa identified in Burgess *et al*. (2012) with one representative on NCBI.

Table S4: Re-analysis of the ZAV archaeal clone library from Burgess *et al.*
This table contains a comparison of the re-analysed archaeal clone library in Zavarzin Spring (ZAV) to the values reported in Burgess *et al*. [11] using modern databases.

Table S5: Re-analysis of the ARK bacterial clone library from Burgess *et al*.
This table contains a comparison of the re-analysed bacterial clone library in Arkashin Schurf (ARK) to the values reported in Burgess *et al*. [11] using modern databases.

Table S6: Re-analysis of the ZAV bacterial clone library from Burgess *et al.*
This table contains a comparison of the re-analysed bacterial clone library in Zavarzin Spring (ZAV) to the values reported in Burgess *et al*. [11] using modern databases.

Table S7: Presence / Absence tables of inferred phyla observed in ARK between metagenome assembled genomes and different sequencing methods.
This table depicts the presence (+) and absence (-) of phyla observed in Arkashin Schurf (ARK) across the metagenome assembled genomes (MAGs), the Ribosomal Database Project v. 11.5 inferred taxonomy of the 16S rRNA gene Sanger clone libraries prepared by Burgess *et al.* [11], the Kaiju v. 1.6.2 inferred taxonomy for the Sanger metagenomes prepared by TIGR and the Kaiju v. 1.6.2 inferred taxonomy for the Solexa reads which were later assembled to bin the MAGs.

Table S8: Presence / Absence tables of inferred phyla observed in ZAV between metagenome assembled genomes and different sequencing methods.
This table depicts the presence (+) and absence (-) of phyla observed in Zavarzin Spring (ZAV) across the metagenome assembled genomes (MAGs), the Ribosomal Database Project v. 11.5 inferred taxonomy of the 16S rRNA gene Sanger clone libraries prepared by Burgess *et al.* [11], the Kaiju v. 1.6.2 inferred taxonomy for the Sanger metagenomes prepared by TIGR and the Kaiju v. 1.6.2 inferred taxonomy for the Solexa reads which were later assembled to bin the MAGs.

Table S9: Pairwise average nucleotide identities for the genera *Desulfurella.*
This table contains the pairwise average nucleotide identities (ANI) values calculated to compare putative *Desulfurella* metagenome-assembled genome (MAGs) from both pools (Arkashin Schurf ARK-08 and Zavarzin Spring ZAV-10) to each other and to available reference genomes. ANI values > 95% are indicated in bold.

Table S10: Pairwise average nucleotide identities for the genera *Sulfurihydrogenibium.*
This table contains the pairwise average nucleotide identities (ANI) values calculated to compare putative *Sulfurihydrogenibium* metagenome-assembled genome (MAGs) from both pools (Arkashin Schurf ARK-13 and Zavarzin Spring ZAV-16) to each other and to available reference genomes. ANI values > 95% are indicated in bold.

Table S11: Predicted arsenic gene pathways in Arkashin Schurf and Zavarzin Spring.

Thirteen homologs of genes involved in the biogeochemical cycle of arsenic according to Zhu *et al*. (2017) [12] were found in the two pools. COG - IDs = IDs of archaeal/bacterial Clusters of Orthologous Groups, ARK (n genes) = number of homologous genes found in 16 bins in Arkashin Schurf and in 20 bins in Zavarzin Spring (ZAV). The last column (Metabolism) indicates whether a predicted gene function is part of an active efflux pump or involved in an arsenic metabolism.

Table S12: Recovery of predicted arsenic gene pathways using Hidden Markov Models (HMMs) for homologous protein sequence classification in Arkashin Schurf and Zavarzin Spring.
Genes involved in the biogeochemical cycle of arsenic were retrieved from Zhu *et al*. (2017) [12]. The total number of complete homologous protein sequences are given for all open reading frames (ORFs) in Arkashin Schurf (ARK) and Zavarzin Spring (ZAV). We also report if homologous genes could be assigned to metagenomic assembled genomes (MAGs).

Table S13: Homologs of predicted KEGG pathways that were found in one pool exclusively.
Shown are all complete KEGG pathways; *i.e.*, gene pathways of which all genes (blocks) were represented that were found in one pool but not in the other; Arkashin Schurf (ARK) and Zavarzin Spring (ZAV). For each pathway its KEGG-ID, name and pathway module are given. Presence of pathways was predicted based on the retrieval of homologous genes.

Table S14: Complete carbohydrate and lipid metabolism KEGG pathways that were predicted to be present in both pools based on the recovery of putatively homologous genes.
Shown are all complete KEGG pathways; *i.e.*, gene pathways of which all genes (blocks) were present with several copies (n > 14 to 1,134) in both pools (Arkashin Schurf and Zavarzin Spring). Presence of pathways was predicted based on the retrieval of homologous genes.

Table S15: Complete environmental information processing KEGG pathways that were predicted to be present in both pools based on the recovery of putatively homologous genes.
Shown are all complete KEGG pathways; *i.e*., gene pathways of which all genes (blocks) were represented that were found in both pools; Arkashin Schurf (ARK) and Zavarzin Spring (ZAV). For each pathway its KEGG-ID, name and pathway module are given. In total there were 78 complete environmental information processing KEGG pathways. For clarity, only mineral and organic ion transport, drug resistance, and bacterial secretion systems are shown. Presence of pathways was predicted based on the retrieval of homologous genes.

Table S16: Exhaustive list of homologs of KEGG pathways in ARK and ZAV.
Kyoto Encyclopedia of Genes and Genomes (KEGG) orthologies were downloaded from GhostKOALA [13] following the workflow for anvi'o by Elaina Graham (as described in http://merenlab.org/2018/01/17/importing-ghostkoala-annotations/). All predicted KEGG pathways including the number of different versions and completeness in both pools are shown.

Figure S1: Placement of the rest of our MAGs into their phylogenetic context.
Taxonomy of our MAGs (metagenome-assembled genomes) was refined by placing them into a phylogenetic tree using PhyloSift v. 1.0.1 with its updated markers database for the alignment and RAxML v. 8.2.10 on the CIPRES web server for the tree inference (Fig. 2 in main manuscript). This tree includes our 36 MAGs (red dots), genomes of all taxa previously identified by Burgess *et al*. (2012) with complete genomes available on NCBI (n = 148; [14]), and 3,102 archaeal (yellow) and bacterial (grey) genomes previously used in Hug *et al*. (2016; [15–17]). The complete tree in newick format and its alignment of 37 concatenated marker genes can be found on figshare [4,7]. Branches with MAGs found in Arkashin Schurf (ARK) and Zavarzin Spring (ZAV) are enlarged (orange nodes). Blue: taxa from Burgess *et al.* (2012), black: taxa from Hug *et al*. (2016). GCA IDs from

NCBI are shown for the closest neighbours of our MAGs. a) Acidobacteriales and Thermodesulfovibrio; b) Sphingobacteriales; and c) Thermodesulfobacteria.

Figure S1

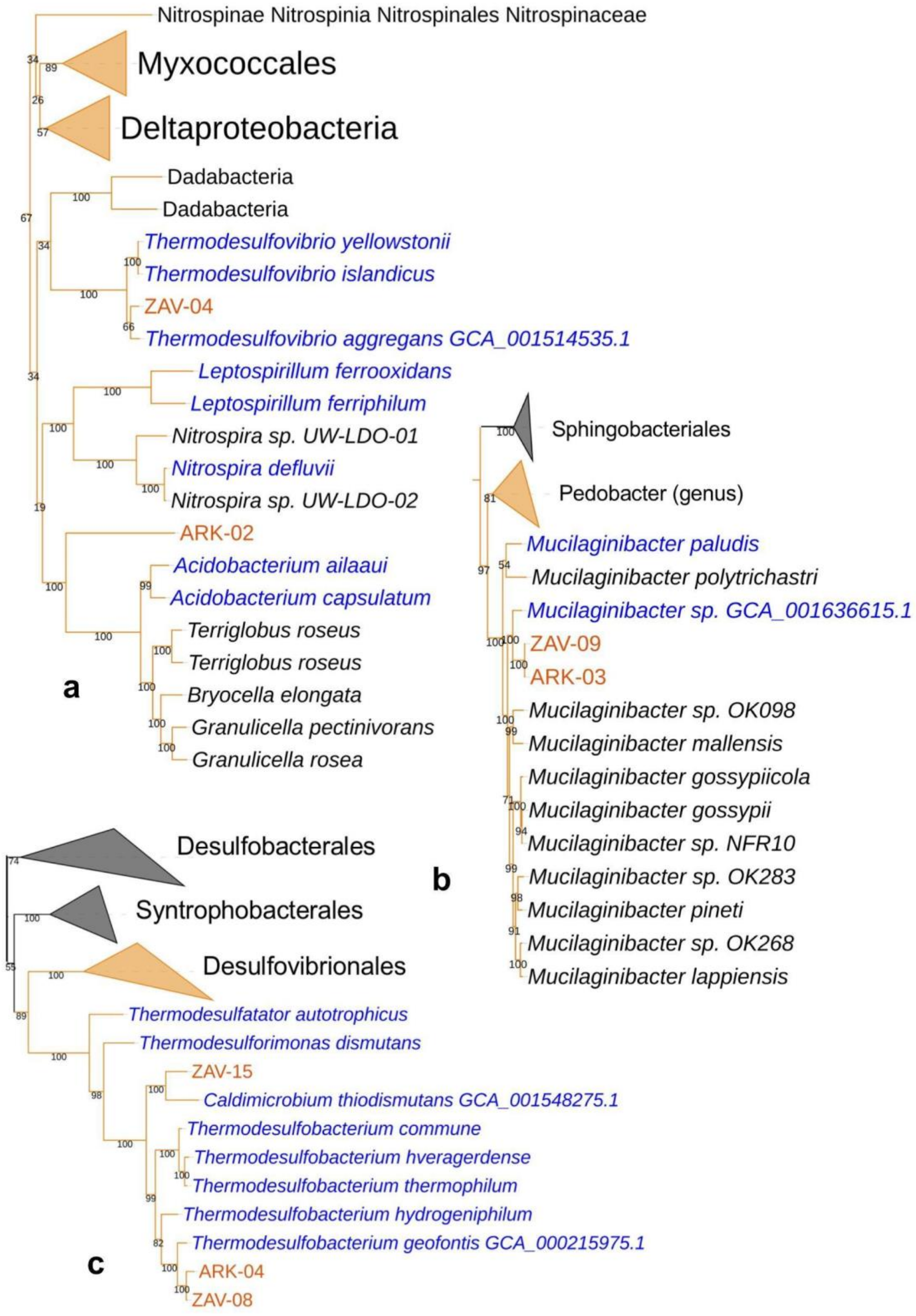
Nitrospinae Nitrospinia Nitrospinales Nitrospinaceae
Myxococcales
Deltaproteobacteria
Dadabacteria
Dadabacteria
Thermodesulfovibrio yellowstonii
Thermodesulfovibrio islandicus
ZAV-04
Thermodesulfovibrio aggregans GCA_001514535.1
Leptospirillum ferrooxidans
Leptospirillum ferriphilum
Nitrospira sp. UW-LDO-01
Nitrospira defluvii
Nitrospira sp. UW-LDO-02
ARK-02
Acidobacterium ailaaui
Acidobacterium capsulatum
Terriglobus roseus
Terriglobus roseus
Bryocella elongata
Granulicella pectinivorans
Granulicella rosea
a
Sphingobacteriales
Pedobacter (genus)
Mucilaginibacter paludis
Mucilaginibacter polytrichastri
Mucilaginibacter sp. GCA_001636615.1
ZAV-09
ARK-03
Mucilaginibacter sp. OK098
Mucilaginibacter mallensis
Mucilaginibacter gossypiicola
Mucilaginibacter gossypii
Mucilaginibacter sp. NFR10
Mucilaginibacter sp. OK283
Mucilaginibacter pineti
Mucilaginibacter sp. OK268
Mucilaginibacter lappiensis
b
Desulfobacterales
Syntrophobacterales
Desulfovibrionales
Thermodesulfatator autotrophicus
Thermodesulforimonas dismutans
ZAV-15
Caldimicrobium thiodismutans GCA_001548275.1
Thermodesulfobacterium commune
Thermodesulfobacterium hveragerdense
Thermodesulfobacterium thermophilum
Thermodesulfobacterium hydrogeniphilum
Thermodesulfobacterium geofontis GCA_000215975.1
ARK-04
ZAV-08
c

Figure S2: Metabolic overview of ARK-15 and ZAV-18

Fasta files for a) ARK-15 (*Aciduliprofundum* sp.) and b) ZAV-18 (*Korarchaeota* sp.) were uploaded to RAST [18] and visualized through its SEED [18] viewer. Features are colored according to functional categories, and numbers of different genes in each category are given in parentheses. Pathways discussed in the manuscript are expanded for ZAV-18 (b).

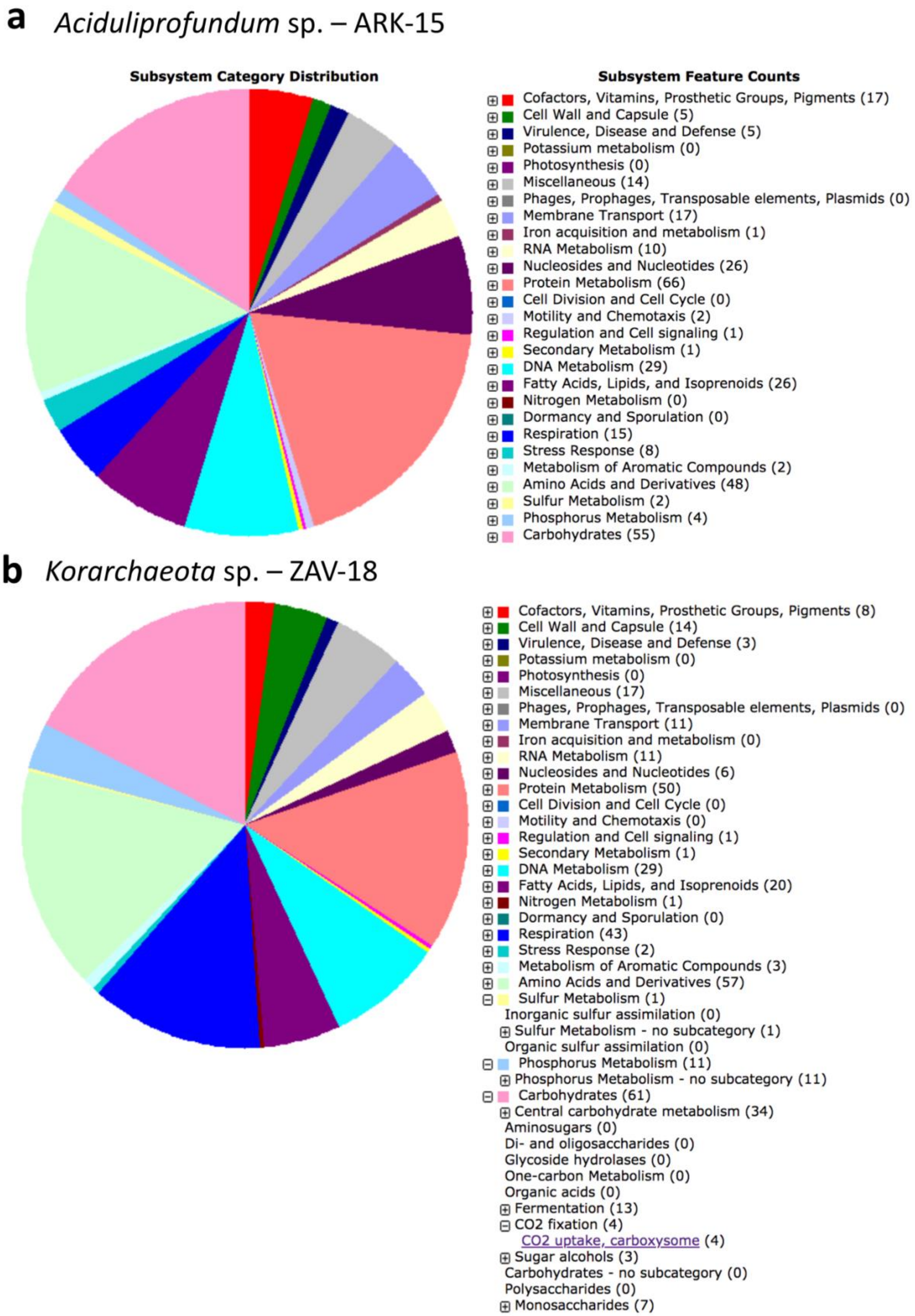
a Aciduliprofundum sp. – ARK-15
Subsystem Category Distribution
Subsystem Feature Counts
Cofactors, Vitamins, Prosthetic Groups, Pigments (17)
Cell Wall and Capsule (5)
Virulence, Disease and Defense (5)
Potassium metabolism (0)
Photosynthesis (0)
Miscellaneous (14)
Phages, Prophages, Transposable elements, Plasmids (0)
Membrane Transport (17)
Iron acquisition and metabolism (1)
RNA Metabolism (10)
Nucleosides and Nucleotides (26)
Protein Metabolism (66)
Cell Division and Cell Cycle (0)
Motility and Chemotaxis (2)
Regulation and Cell signaling (1)
Secondary Metabolism (1)
DNA Metabolism (29)
Fatty Acids, Lipids, and Isoprenoids (26)
Nitrogen Metabolism (0)
Dormancy and Sporulation (0)
Respiration (15)
Stress Response (8)
Metabolism of Aromatic Compounds (2)
Amino Acids and Derivatives (48)
Sulfur Metabolism (2)
Phosphorus Metabolism (4)
Carbohydrates (55)
b Korarchaeota sp. – ZAV-18
Cofactors, Vitamins, Prosthetic Groups, Pigments (8)
Cell Wall and Capsule (14)
Virulence, Disease and Defense (3)
Potassium metabolism (0)
Photosynthesis (0)
Miscellaneous (17)
Phages, Prophages, Transposable elements, Plasmids (0)
Membrane Transport (11)
Iron acquisition and metabolism (0)
RNA Metabolism (11)
Nucleosides and Nucleotides (6)
Protein Metabolism (50)
Cell Division and Cell Cycle (0)
Motility and Chemotaxis (0)
Regulation and Cell signaling (1)
Secondary Metabolism (1)
DNA Metabolism (29)
Fatty Acids, Lipids, and Isoprenoids (20)
Nitrogen Metabolism (1)
Dormancy and Sporulation (0)
Respiration (43)
Stress Response (2)
Metabolism of Aromatic Compounds (3)
Amino Acids and Derivatives (57)
Sulfur Metabolism (1)
Inorganic sulfur assimilation (0)
Sulfur Metabolism - no subcategory (1)
Organic sulfur assimilation (0)
Phosphorus Metabolism (11)
Phosphorus Metabolism - no subcategory (11)
Carbohydrates (61)
Central carbohydrate metabolism (34)
Aminosugars (0)
Di- and oligosaccharides (0)
Glycoside hydrolases (0)
One-carbon Metabolism (0)
Organic acids (0)
Fermentation (13)
CO2 fixation (4)
CO2 uptake, carboxysome (4)
Sugar alcohols (3)
Carbohydrates - no subcategory (0)
Polysaccharides (0)
Monosaccharides (7)

References

1. Eisen, J. A. *et al.* The complete genome sequence of Chlorobium tepidum TLS, a photosynthetic, anaerobic, green-sulfur bacterium. *Proceedings of the National Academy of Sciences* **99**, 9509–9514 (2002).
2. Hug, L. A. *et al.* A new view of the tree of life. *Nat Microbiol* **1**, 16048 (2016).
3. Capella-Gutiérrez, S., Silla-Martínez, J. M. & Gabaldón, T. trimAl: a tool for automated alignment trimming in large-scale phylogenetic analyses. *Bioinformatics* **25**, 1972–1973 (2009).
4. Wilkins, L. G. E., Ettinger, C. L., Jospin, G. & Eisen, J. A. Alignment for tree of life. Figshare. Dataset. doi:10.6084/m9.figshare.6916298.v1
5. Miller, M. A., Pfeiffer, W. & Schwartz, T. Creating the CIPRES Science Gateway for inference of large phylogenetic trees. in *2010 Gateway Computing Environments Workshop (GCE)* (2010). doi:10.1109/gce.2010.5676129
6. Le, S. Q. & Gascuel, O. An improved general amino acid replacement matrix. *Mol. Biol. Evol.* **25**, 1307–1320 (2008).
7. Wilkins, L. G. E., Ettinger, C. L., Jospin, G. & Eisen, J. A. Tree of life - archaea and bacteria from Hug et al.'s tree of life in 2016; MAGs isolated from two hot springs in the Uzon Caldera, Kamchatka, Russia; and all taxa from Burgess et al. (2012) with one representative genome on NCBI (Newick file). Figshare. (2018). doi:10.6084/m9.figshare.6874928.v3
8. Letunic, I. & Bork, P. Interactive tree of life (iTOL) v3: an online tool for the display and annotation of phylogenetic and other trees. *Nucleic Acids Res.* **44**, W242–5 (2016).

9. Ettinger, C. L. Kamchatka Zavarzin Spring Metagenome Analysis Notebook. Figshare. doi:10.6084/m9.figshare.6873743.v1

10. Wilkins, L. G. E. Kamchatka Arkashin Schurf Metagenome Analysis Notebook. Figshare. Code. (2018). doi:10.6084/m9.figshare.6874925.v1

11. Burgess, E. A., Unrine, J. M., Mills, G. L., Romanek, C. S. & Wiegel, J. Comparative geochemical and microbiological characterization of two thermal pools in the Uzon Caldera, Kamchatka, Russia. *Microb. Ecol.* **63**, 471–489 (2012).

12. Zhu, Y.-G., Xue, X.-M., Kappler, A., Rosen, B. P. & Meharg, A. A. Linking Genes to Microbial Biogeochemical Cycling: Lessons from Arsenic. *Environ. Sci. Technol.* **51**, 7326–7339 (2017).

13. Kanehisa, M., Sato, Y. & Morishima, K. BlastKOALA and GhostKOALA: KEGG Tools for Functional Characterization of Genome and Metagenome Sequences. *J. Mol. Biol.* **428**, 726–731 (2016).

14. Wilkins, L. G. E., Ettinger, C. L., Jospin, G. & Eisen, J. A. Genomes from Burgess et al. (2012) from two hot springs in Kamchatka, Russia. Figshare. doi:10.6084/m9.figshare.6863798.v1

15. Wilkins, L. G. E., Ettinger, C. L., Jospin, G. & Eisen, J. A. Genomes in the tree of life of Hug et al. (2016) - part I. Figshare. doi:10.6084/m9.figshare.6863594.v1

16. Wilkins, L. G. E., Ettinger, C. L., Jospin, G. & Eisen, J. A. Genomes in the tree of life of Hug et al. (2016) - part II. Figshare. doi:10.6084/m9.figshare.6863744.v2

17. Wilkins, L. G. E., Ettinger, C. L., Jospin, G. & Eisen, J. A. Genomes in the tree of life of Hug et al. (2016) - manually downloaded. Figshare. doi:10.6084/m9.figshare.6863813.v1

18. Aziz, R. K. *et al.* The RAST Server: rapid annotations using subsystems technology. *BMC Genomics* **9**, 75 (2008).
